# Novel Fiber Optic Sensor Probe with a Pair of Highly Reflected Connectors and a Vessel of Water Absorption Material for Water Leak Detection

**DOI:** 10.3390/s120810906

**Published:** 2012-08-07

**Authors:** Tae-Sik Cho, Ki-Sun Choi, Dae-Cheol Seo, Il-Bum Kwon, Jung-Ryul Lee

**Affiliations:** 1 Center for Safety Measurement, Korea Research Institute of Standards and Science, 1 Doryong-dong Yuseong-gu, Daejeon 305-340, Korea; E-Mails: tscho@kriss.re.kr (T.-S.C.); kschoi@kriss.re.kr (K.-S.C.); dcseo@kriss.re.kr (D.-C.S.); 2 Department of Aerospace Engineering, Jeonbuk National University, Jeonju 561-756, Korea; E-Mail: leejrr@jbnu.ac.kr

**Keywords:** water leak detection, highly reflected connector, reference connector, sensing connector, sensor probe, water combination soil, vessel

## Abstract

The use of a fiber optic quasi-distributed sensing technique for detecting the location and severity of water leakage is suggested. A novel fiber optic sensor probe is devised with a vessel of water absorption material called as water combination soil (WCS) located between two highly reflected connectors: one is a reference connector and the other is a sensing connector. In this study, the sensing output is calculated from the reflected light signals of the two connectors. The first reflected light signal is a reference and the second is a sensing signal which is attenuated by the optical fiber bending loss due to the WCS expansion absorbing water. Also, the bending loss of each sensor probe is determined by referring to the total number of sensor probes and the total power budget of an entire system. We have investigated several probe characteristics to show the design feasibility of the novel fiber sensor probe. The effects of vessel sizes of the probes on the water detection sensitivity are studied. The largest vessel probe provides the highest sensitivity of 0.267 dB/mL, while the smallest shows relatively low sensitivity of 0.067 dB/mL, and unstable response. The sensor probe with a high output value provides a high sensitivity with various detection levels while the number of total installable sensor probes decreases.

## 1. Introduction

Fiber optic sensors can be used to monitor large structures such as bridges, dams, pipelines, *etc*. They can also provide long distance sensing solutions with many measurement points through one optical fiber line in a technique known as distributed sensing, which is very unique for fiber optic sensing technologies [1]. Fully distributed sensing techniques are usually implemented by a sensing optical fiber which can sense some external disturbance influencing any location on the fiber. In general, the fully distributed sensing fiber cables are usually expensive because the fibers should be specially fabricated to sense external disturbances [2–4]. However, the quasi-distributed sensing techniques can be economically implemented with point sensing probes installed on conventional optical fibers even though the number of sensing points on each probe are limited.

For detecting water leakage, various fiber optic sensors have been developed by utilizing hydrogels [5], polyacrylic acid polymers [6], and the mechanical macro-bending technique [7]. These can be implemented by a configuration of quasi-distributed sensing techniques for multi-sensor systems. In the water leak detection methods, swellable material techniques have been employed due to their simple detection scheme since they absorb water and result in a bending loss due to their expansion [8,9]. For detecting sensing locations and levels of events, an optical time domain reflection (OTDR) technique has been widely employed [10–13]. OTDR measurements allow locating and detecting water leak events from the sharp drops of backscattered light [14]. However, these light scattered techniques based on OTDR should usually detect small and short backscattered lights which limit the dynamic ranges of sensors [15]. The small dynamic range causes a reduction of the number and sensitivity of installable sensors. Therefore, it is necessary to develop new sensing techniques with large dynamic ranges to detect the location and severity of water leakages.

In this paper, a novel fiber optic sensor probe based on an OTDR measurement is devised using a pair of highly reflected connectors on an optical fiber in order to enhance reflected signals and a vessel of water absorption material in order to detect water leakage. The bending loss of a sensor head is studied according to the amount of water leakage. Three types of sensor probes, having different vessel sizes, are fabricated to study the responsivity to water absorption. Two sensor probes of the same type are connected through one optical fiber line to investigate the characteristics of multiplexing operation. Moreover, a link budget is simply analyzed to investigate how an entire sensor system can be designed to satisfy the demands of users.

## 2. Sensor Principle

### 2.1. Principle of a Multiple Optical Loss Based Fiber Optic Sensor Using OTDR

**Figure 1.**
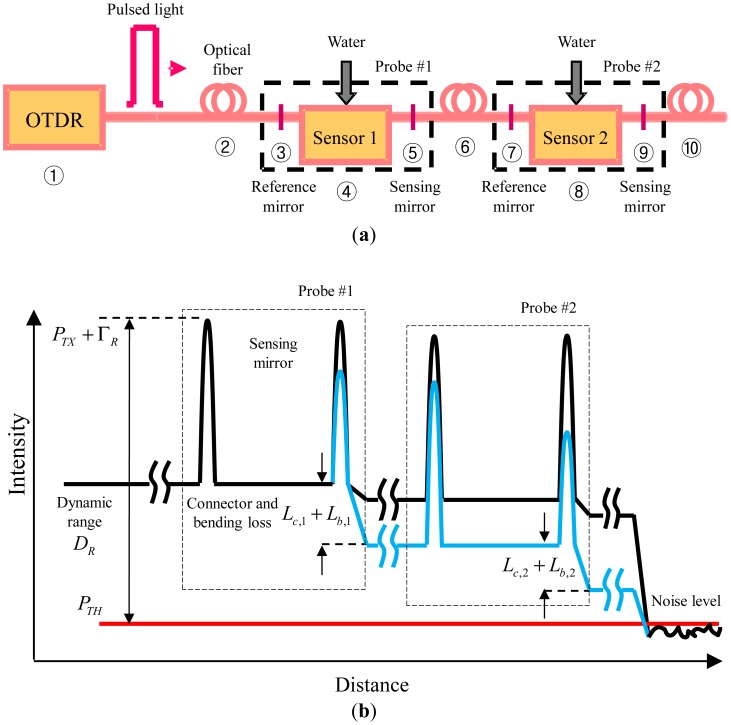
Schematic diagram of MOL-FOS using OTDR. (**a**) Configuration of MOL-FOS, WCS: Water combination soil; (**b**) The intensity of the received light.

A multiplexed optical loss based fiber optic sensor (MOL-FOS) using OTDR with new sensor probes for sensing reflected signals and measuring a position as shown in Figure 1. The sensor probe has a pair of highly reflected connectors: the first one is a reference connector and the second one is a sensing connector as shown in Figure 1(a). If the pulsed light meets the reference connector and the sensing connector, then some portion of the pulsed light shall be reflected at the connectors. The rest of the pulsed light can travel through the optical fiber line to operate other sensor probes. Figure 1(b) shows the reflected lights from two connectors. If water leakage induces the bending of the optical fiber between the reference connector and the sensing connector, then the bending causes some optical loss between the two connectors (blue line). This optical loss can be detected by the reflected light decrease of the sensing connector. Also, when the light is decreased at the front of the reference connector, then the reflected light signal can indicate the decrease of the effective light intensity. Therefore, if the relation between the water leakage and the change of the sensing signal can be found, then the water leakage can be detected from this fiber optic sensor. It is noteworthy that our sensor probes are a kind of leak detector. Thus, we deploy the sensors in the field and check whether the event occurs or not. If the event happens, the sensors should be exchanged for new ones.

### 2.2. Principle of a Fiber Optic Sensor Probe for Water Leak Detection

**Figure 2.**
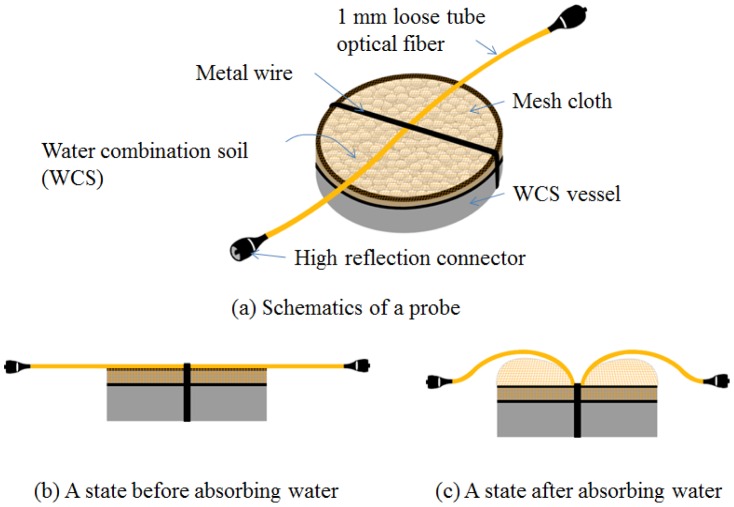
Configuration of a probe for water leak detection.

**Figure 3.**
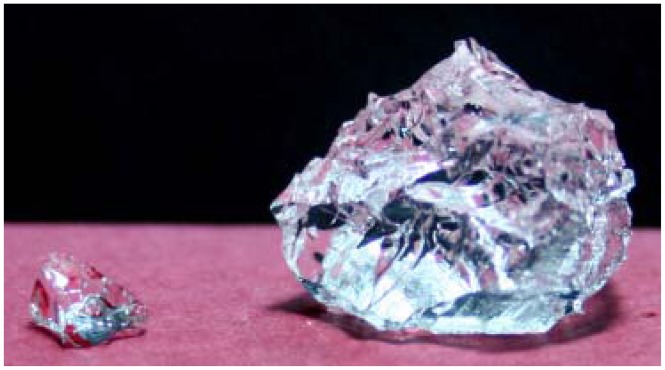
Feature of water combination soil (WCS).

A fiber optic sensor probe is designed to detect water leakage using a pair of high reflection connectors and a vessel with water combination soil (WCS) as shown in Figure 2(a). The chemical elements of WCS are acrylate polymers with an absorbance rate of 30 seconds. This probe has a vessel full of WCS and a mesh cloth and a metal wire on the opening part of the vessel. An optical fiber is located on the mesh cloth under the metal wire of the vessel. When the probe is in the state before absorbing water, the optical fiber is not bent, as shown in Figure 2(b). The volume of WCS will expand about 20 times the initial volume when it absorbs water as shown in Figure 3.

This WCS material becomes jellylike when it absorbs water. The holes of the mesh cloth should be small enough to prevent the WCS from falling out of the vessel. However, water should be able to enter this vessel and contact the WCS. If the WCS meets water, then it will expand through the opening part of the vessel. Then, the mesh cloth will expand by the expansion of WCS, and the optical fiber will be bent by the wire preventing this expansion as shown in Figure 2(c). The mesh used is a stocking material which consists of nylon and polyurethane. The breaking strength per 0.01 mm is 4 kg, and the mesh size is less than 1 mm [16]. As a result of this bending, the sensing signal of the sensor probe will be changed. Therefore, this signal change can indicate the water leakage. In order to define the output (*S_k_*) at the k^th^ sensor probe, we employ an equation using the ratio between the reference signal and the sensing signal as follows:

(1) $$S_{k}=-10\log_{10}\left(\frac{I_{s,k}/I_{s0}}{I_{r,k}/I_{r0}}\right)$$

where *I_s0_* and *I_r0_* are the intensities of a sensing and reference signal at the initial state of the sensor probe, and *I_s,k_* and *I_r,k_* are the intensities of the sensing signal and the reference signal respectively at the present time in the k^th^ sensor probe. As shown in Equation (1), the sensor output, *S*, will be zero at the initial state or there is no water leak. However, if some water gets into the probe, then the sensor output will increase. This sensor probe having special two connectors is novel because the signal output, S_k_, can be optimized by the reflection applying the connectors having optimal reflection rate. In other words, we can adjust the magnitude of the signal output of each sensor probe by controlling the reflection rate of the connectors.

### 2.3. Dynamic Range of MOL-FOS

In order to design the MOL-FOS with OTDR, the total light power should be well distributed to each sensor probe. The dynamic range of MOL-FOS can be simply evaluated with the peak power of a reflected light and a threshold power. By calculating the dynamic range, the number of installable sensor probes is calculated as follows:

(2) $$D_{R}=\Gamma_{T}+\Gamma_{R}$$

(3) $$D_{R}-\alpha \cdot L-\sum_{k=1}^{N_{S}}(L_{c,k}+L_{b,k})>0$$

(4) $$N_{s}<\frac{D_{R}-\alpha \cdot L}{L_{c}+L_{b}}\;\text{for}\;L_{c,k}=L_{c}\;\text{and}\;L_{b,k}=L_{b}$$

where *D_R_* is a dynamic range (dB) of a sensor system, Γ*_T_*(=10log_10_*P_TX_*/*P_TH_*) is the rate of a transmitted laser power *P_TX_* and a threshold power *P_TH_* for a leak detection. Γ*_R_*(=10log_10_*P_R_*/*P_TX_*) is the reflection rate with respect to a transmitted power (dB) where *P_R_* is the reflected power. *N_S_* is the number of required sensor probes, *α* is a fiber loss, *L* is a fiber length, *L_c,k_* and *L_b,k_* are the k^th^ connector loss and bending loss due to water leak. It is noteworthy that the sensitivity of a sensor probe for water leaks is related with *L_b,k_*. Thus, the high sensitivity of a sensor results in a large *L_b,k_*. By considering the dynamic range and the k^th^ sensor loss, including connector loss and bending loss, the effective dynamic ranges of multiplexing sensor probes can be evaluated from Equation (3). If we assume that all sensor probe loss properties are same, the maximum number of installable sensor probes is described in Equation (4).

## 3. Water Leak Detection Experiments

### 3.1. Experimental Setup

**Figure 4.**
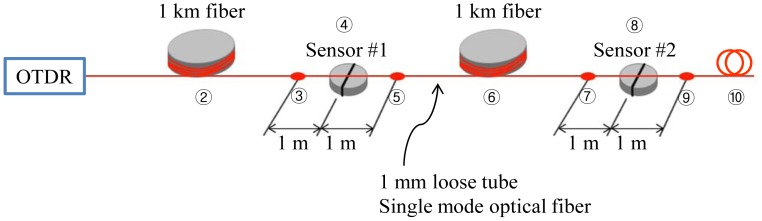
Experimental setup of MOL-FOS for water leak detection.

The experimental setup of MOL-FOS is described in Figure 4. Two probes are multiplexed on one optical fiber line. The reference and sensing connectors, which are conventional FC/PC type optical connectors having high contact surface roughness to produce a reflectivity that is higher than in the conventional ones, are installed at a location 2 m apart from the vessel of each sensor probe, respectively. The light source is operated with a pulse width of 10 ns which provides 1 m resolution as described in Table 1.

**Figure 5.**
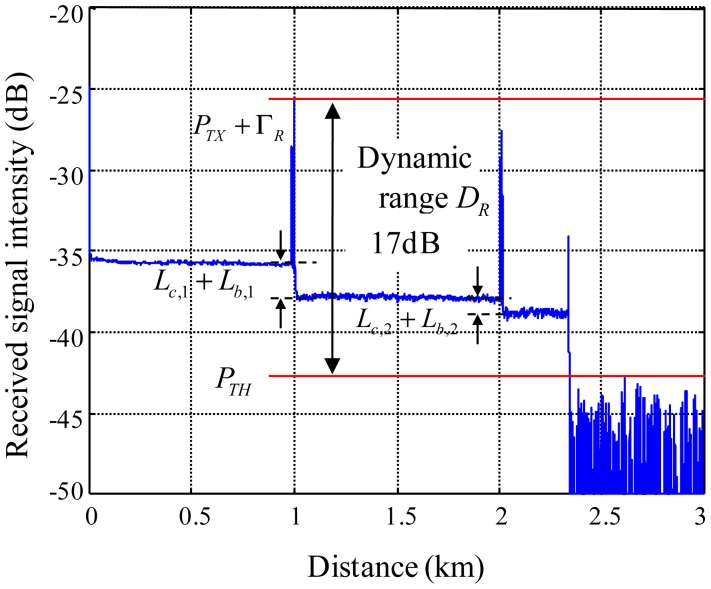
Dynamic range of MOL-FOS.

Figure 5 shows reflected signals acquired from the MOL-FOS. As shown in the figure, the dynamic range is around 17 dB. The level of a backscattered signal power is −36 dBm. *P_TX_* + Γ*_R_* is −26 dBm, *P_TH_* is −43 dBm, and *L_c_*,_1_ and *L_c_*,_2_ is 2.5 and 1 dB, respectively. If we don't employ a sensing connector at the sensor probes, the dynamic range will be 7 dB (= −36 dB + 43 dB) since the reflected signal from a sensing connector will disappear. If we set *L_c_* and *L_b_* to 2 and 2 dB, and optical fiber loss is less than 1 dB, then the number of installable sensor probes will be 4. The number of sensor probes that can be installed on one optical fiber line, 4, is noteworthy. If it is necessary to provide sensor probes higher dynamic ranges, then the total number of sensors on one optical fiber line should be decreased to provide more power to each sensor probe.

**Figure 6.**
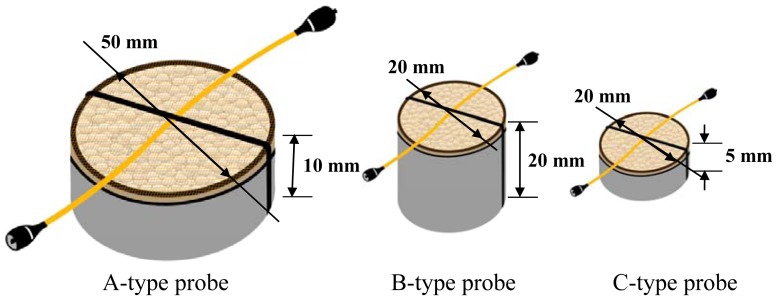
Fiber optic sensor probes using three types of vessels.

In order to study the performance of the devised sensor probe, three types of sensor probes A, B and C, are fabricated using three vessels, as shown in Figure 6. The vessel of each sensor probe is filled with WCS of about 2 mm diameter and having a single mode optical fiber with 1 mm loose tube and a metal wire of the diameter of 1 mm, as shown in Figure 2. The vessels of the probes are also closed with mesh cloths having many holes of less than 1 mm in order to keep WCS and water passing through in the vessels. For making the bending loss of the fiber at the WCS expansion condition, the optical fiber is located on the mesh cloth and under the metal wire and with the crossing direction of the metal wire. The power loss due to connector reflection of each sensor probe is controlled at about 2.5 dB by tuning the reflectivity of the optical connectors.

### 3.2. Response of Single Fiber Optic Sensor Probe

**Figure 7.**
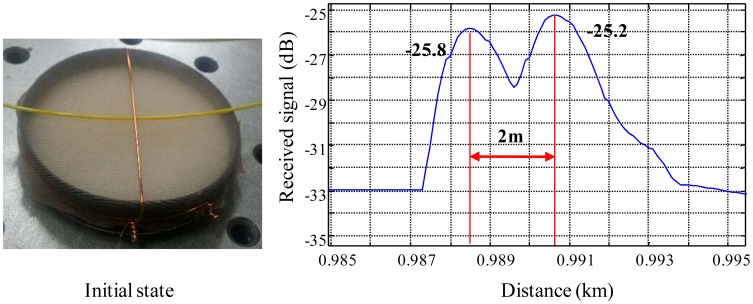
Initial state and received signal of a sensor probe.

We investigate, first, the tension effect of the metal wire because it may influence the bending loss before the response test of the sensor probes. Fortunately, the force of the WCS expansion is not enough to bend the metal wire because the wire is strongly tightened and very stiff. Thus, we neglect the wire tension effect on the bending loss due to the WCS expansion in this study. In addition, the single mode fiber (SMF) is tightly jacketed with 1 mm-diameter plastic tube. If we assume that bare fibers are employed, the bending loss will be much larger than that of the 1 mm-loose tube SMF because 1 mm plastic jacketed SMF is stiffer than bare fibers. In order to study the response of a sensor probe, at first, we test a single sensor probe with the sensor system. In the result of this test, the reflected signals from the reference connector and the sensing connector are −25.8 and −25.2 dB, respectively, at the initial state of no-water absorption shown in Figure 7. We can know that these two reflections are located 2 m apart from each other.

**Figure 8.**
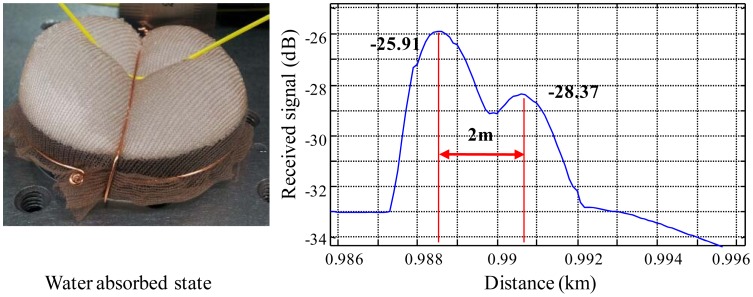
Absorbed state and received signal of a sensor probe.

In Figure 8, the signal of the sensing connector is decreased about 3 dB because of the bending loss caused by the expansion of WCS absorbing water. The detection sensitivity of the sensor is governed by the relation between bending loss and water absorbance. Therefore, it is noteworthy that the maximum bending loss, sensor margin, of each sensor probe should be determined by considering the total power margin in order to operate the entire sensor probes through one optical fiber line. If it assumes that the bending loss is quite high even for absorbing a small amount of water, then the sensor probe is too sensitive. However, although a severe expansion of WCS material occurs, the bending loss is not fully developed because the water absorbing WCS becomes jellylike after absorbing water within several minutes in this experiment. Therefore, the relation of the bending loss with absorbing water should be carefully studied to design the whole multiplexing sensor system.

Generally, the bending of SMF will be directly affected by the metal wire diameter when the optical fiber is bent on the surface of the wire by a sufficient force. However, the bending radius of the optical fiber in our sensor probe is more influenced by the expansion force of WCS. As shown in Figure 8, the optical fiber is bent with a relatively large curvature as compared with the metal wire diameter.

Therefore, the bending of the fiber will not affected by the metal wires having the diameter of less than 0.6 mm of the present metal wire. Therefore, it is no need to be concerned about the effect of the diameter of the metal wire on the bending loss of SMF in our sensor probes. Also, our sensor probes are designed as simple water leakage detection tools. This means that they are to be exchanged after a water detection event. Thus, we will focus on studying the effect of several vessel types and water absorption in this study.

### 3.3. Response of Multiple Fiber Optic Sensor Probes

Two sensor probes are multiplexed through on optical fiber line as shown in Figure 4. Two pairs of sensing and reference connectors are connected and the fiber bending effects studied. The fiber bending loss between these two connectors is investigated by winding the fiber one-turn on a cylindrical mandrel having several diameters.

**Figure 9.**
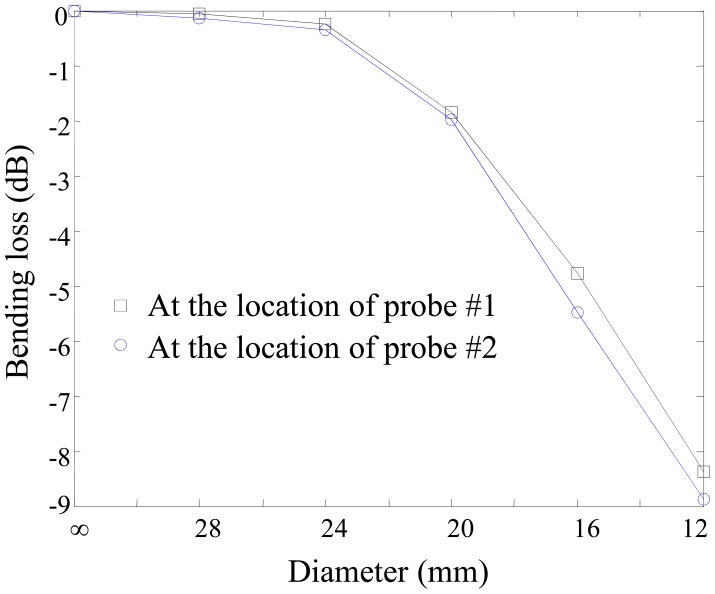
Bending loss according to winding one turn of an optical fiber, SMF-28, on a mandrel with various diameters.

**Figure 10.**
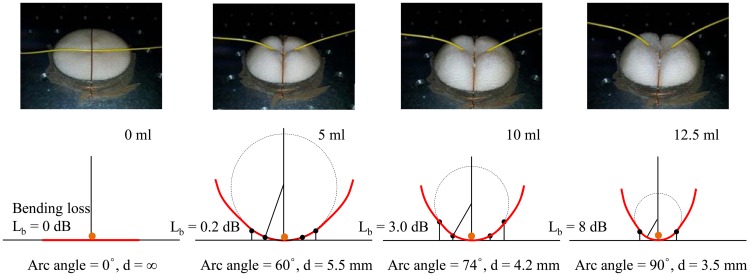
Bending loss according to bending diameters and arc angles of an optical fiber (SMF-28).

As shown in Figure 9, the bending losses at the location of two probes are almost same and the differences are less than 1 dB in the bending diameter range of 12–24 mm. However the bending losses are gradually increased according to the decrease of the bending diameter. In Figure 9, the curves show the bending loss effect due to the bending diameter. The bending loss is generally directly related to the radius of curvature and the arc length [17]. The output power of the sensor probe considering bending loss, *P_out_*, will be as follows:

(5) $$P_{\text{out}}=C_{1}P_{in}\exp(-C_{2}\gamma s)$$

where C_1_ and C_2_ are constants that depend on the dimensions of the waveguide, and on the shape of the optical mode. *P_in_* is an input power, *γ* is the radius of curvature, *s* is an arc length. By employing an arc angle, we can evaluate the radius of curvature. In order to investigate the radius and arc angle of our sensor probe, we measure the vertical location of the optical fiber and use trigonometric relations to determine the bending radius, as shown in Figure 10.

To confirm the evaluated diameter and measured bending loss, we utilize a cylindrical mandrel with various diameters. These diameters shown in Figure 10 are matched with the measured diameters in Figure 9. In order to evaluate the diameter of the cylindrical mandrel case in Figure 9, the coefficient considering the arc angle should be multiplied by the diameters in Figure 10. The coefficient can be easily obtained from Equation (5). In the 3.0 dB bending loss case, the diameter in the cylindrical mandrel case will be about 20. 4 mm (=4.2 mm × 360 degree/74 degree) since the bending loss in Figure 9 is measured under winding an optical fiber onto the cylindrical mandrel once.

**Figure 11.**
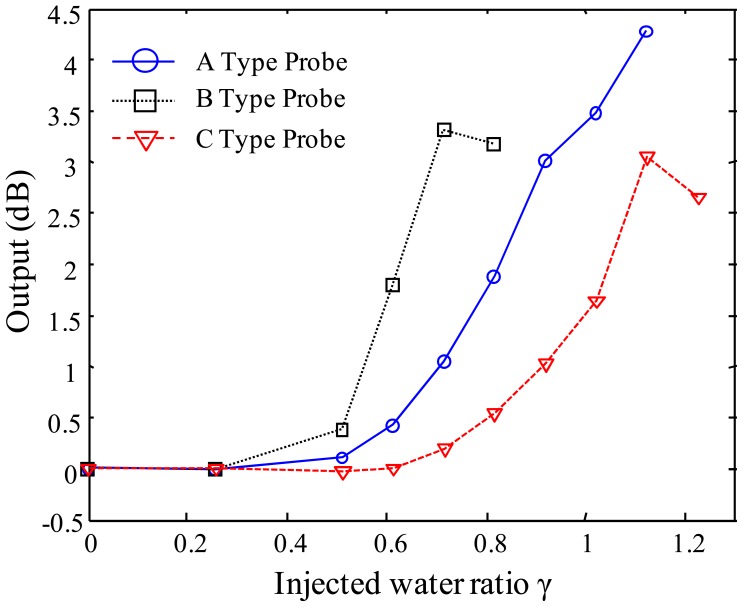
Sensor outputs according to injected water ratio of three types of probes.

After studying the bending losses, the vessels with WCS are designed to ensure the sensor probe responses by having bending diameters of less than 24 mm according to the water absorption. Then, three sensor probes, A, B and C-type, are prepared to study the size effect of the probe shown in Figure 6. An amount of water (12 mL) is injected in the vessel by an injector at regular time intervals, about 5 minutes. The sensor outputs of these probes are investigated according to the amount of water absorption as shown in Figure 11. In the figure, the injected water ratio, *γ*, is defined as the volume of water normalized with respect to the volume of its own vessel. When more and more water is injected in the vessel, B-type probe shows the fastest response to water injection. A and B-type probes are starting to be responsive at the injected water ratio of 0.5. However, C-type probe is responsive from the injected water ratio of 0.7. Also, the B and C-type probes show some small declines in the sensor outputs after the injected water ratios of 0.7 and 1.1, respectively. These can be explained by the fact that the WCS material becomes jellylike after absorbing water. Therefore, it can be concluded that WCS material can't bend the optical fiber any even though more water is injected into the vessel. A-type probe might also show the same signal decline if some more water were provided.

**Figure 12.**
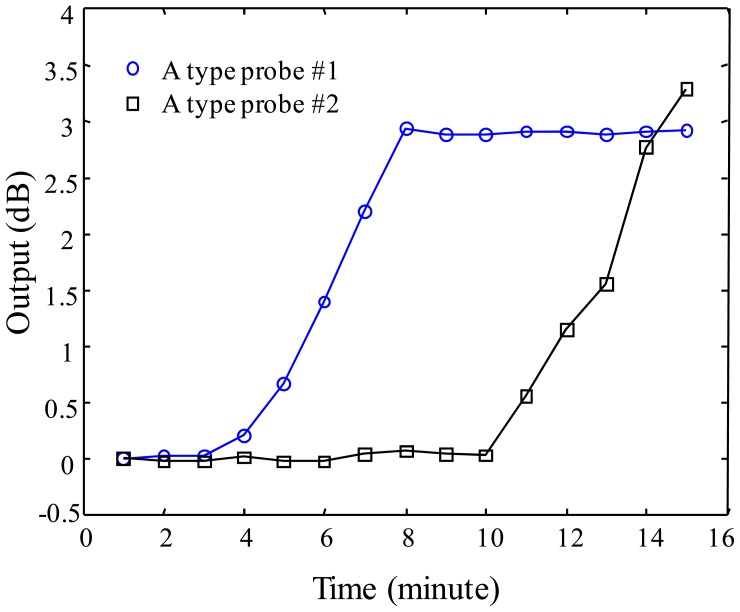
Sensor output of A-type probe as a function of water injection time.

**Figure 13.**
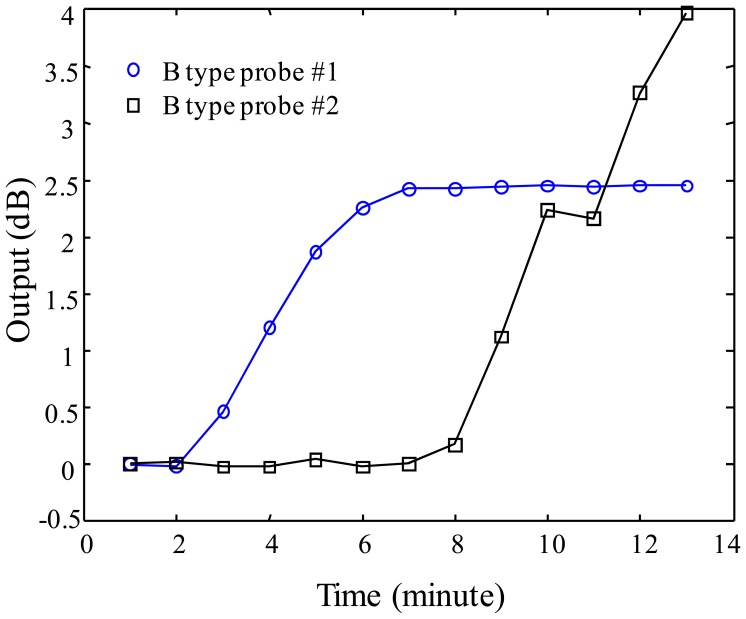
Sensor output of B-type as a function of water injection time.

**Figure 14.**
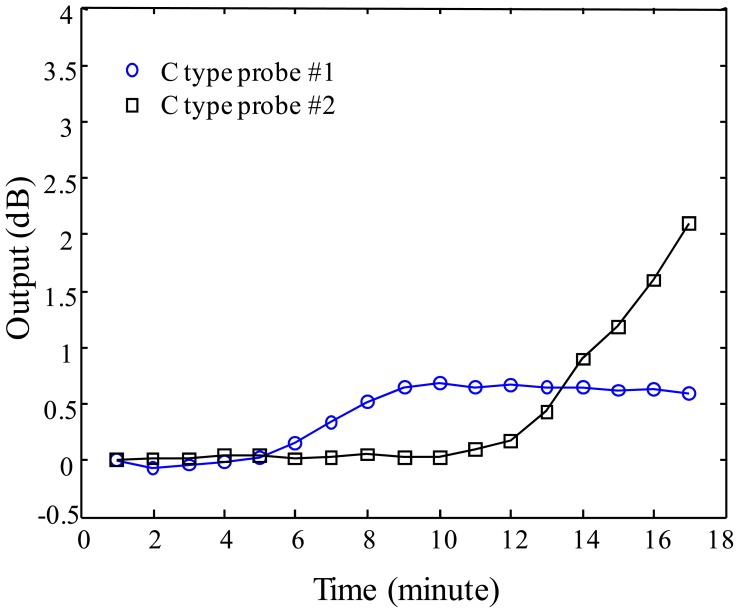
Sensor output of C-type as a function of water injection time.

For investigating the multiplexing operation of two sensor probes, three different configurations are prepared with A-A, B-B, C-C type probes connected on one optical fiber line. The experiment is performed by dropping water on the vessel of sensor probe #1 at time intervals of 1 minute eight times and, after dropping water on the vessel of the probe #1, water continuously is dropped on the vessel of the probe #2 at the time interval of 1 minute eight times. The responses of A-A, B-B, and C-C probe multiplexing are shown in Figures 11–13 for the three probe types. Figure 11 shows the result of sensor output *S* for the multiplexing response of two A-type probes. As shown in the graph, the probe #1 starts to respond gradually from 3 minutes to 8 minutes. The response rate of the probe #1 is determined as 0.8 dB/min in the linear response range of the graph, then, the sensitivity of the probe #1 can be found as 0.267 dB/mL which is calculated by using 3 mL of water instead of 1 minute of time. After showing the response of the probe #1, water is dropped on the vessel of the probe #2 at the time of 8 minutes in Figure 12. The response of the probe #2 shows slightly bigger than that of the probe #1. In the meantime, the multiplexing response of two B-type probes is also shown in Figure 13. In this figure, the response rate of probe #1 is 0.7 dB/min, and then, the sensitivity is 0.233 dB/mL. The probe #2 responds slightly quicker than the probe #1, also the response of this probe is bigger than the probe #1 caused from the probe uncertainty. In Figure 13, there is an unexpected point between 10 and 12 min. We think that it comes from the uncertainty of the expansion time of WCS material. Finally, in Figure 14, the response rate of probe #1 is 0.2 dB/min, and the sensitivity is 0.067 dB/mL. However, the response rate of the probe #2 appears to be slightly quicker than that of the probe #1, which is caused by the uncertainty of probe #2.

The sensitivity of the A type probe is almost same as that of the B type probe, however, the C type probe has the smallest sensitivity in this study. This work suggests the idea to develop a novel fiber optic sensor probe using two highly reflected connectors and a vessel of WCS in order to apply the sensors to detect some water leakage by managing the sensitivity of the probe by controlling the bending loss mechanism with WCS and the dynamic range of the probe by controlling the connector reflectivity.

## 4. Conclusions

We have demonstrated a novel fiber optic sensor probe with a pair of highly reflected connectors and a vessel of water absorption material, WCS, for water leak detection. The bending loss of the optical fiber was studied to confirm the sensor response due to water absorption. The sensing output is calculated to obtain the response between the reflected light signals of two connectors and the optical fiber bending loss by the WCS expansion due to water absorption. Also, the dynamic range of each sensor probe can be determined by considering the bending loss of each sensor probe due to WCS expansion. In order to show the sensor characteristics of the novel fiber sensor probes, we fabricated three types of probes (A, B, and C type) and investigated the sensitivity of each probe. The large vessel probes (A, B type) provide consistent responses having sensitivity of about 0.233–0.267 dB/mL while the smallest one shows a relatively low sensitivity of 0.067 dB/mL. In conclusion, by managing the sensitivity of the probe by controlling the bending loss mechanism with WCS and the dynamic range of the probe by controlling the connector reflectivity, we can determine the number of sensors and total optical fiber length in order to design an optimal fiber optic sensor system to meet sensing requirements in the leak detection field.

## Figures and Tables

**Table 1.** Optical fiber and fiber optic sensor system.

**Device**	**Parameter**	**Value**
Fiber	Type Length (km)	1 mm loose tube SMF 1 km 2 ea
Metal wire	Diameter Material	0.6 mm Cu
WCS	Material	Acrilate polymers
Mesh	Material Breaking strength per 0.01 mm Mesh size	Nylon, polyurethane 4 kg Less than 1 mm
OTDR	Laser power Wavelength Pulse width No. of average	0 dBm 1,625 nm 10 ns 10,000
